# Discovery of a series of novel phenylpiperazine derivatives as EGFR TK inhibitors

**DOI:** 10.1038/srep13934

**Published:** 2015-09-09

**Authors:** Juan Sun, Xin-Yi Wang, Peng-Cheng Lv, Hai-Liang Zhu

**Affiliations:** 1 School of Life Sciences, Shandong University of Technology, Zibo, China; 2 State Key Laboratory of Pharmaceutical Biotechnology, Nanjing University, Nanjing 210093, P. R. China

## Abstract

Human epidermal growth factor receptor (EGFR) is an important drug target that plays a fundamental role in signal transduction pathways in oncology. We report herein the discovery of a novel class of phenylpiperazine derivatives with improved potency toward EGFR. The biological activity of compound **3p** as inhibitor of EGFR was further investigated both *in vitro* and *in vivo*. Notably, compound **3p** exhibited an IC_50_ in the nanomolar range in A549 cell cultures and induced a cessation of tumor growth with no toxicity, as determined by loss of body weight and death of treated mice. Compoutational docking studies also showed that compound **3p** has interaction with EGFR key residues in the active site.

Protein kinases regulate many critical cellular processes through protein phosphorylation, such as proliferation, differentiation and DNA damage/repair^1^, and therefore have been proved to be important drug discovery targets^2^,^3^,^4^,^5^. Among these kinases, the family of human epidermal growth factor receptor (EGFR), is an important drug target that play a fundamental role in signal transduction pathways in oncology. Deregulation of the EGFR family pathway by overexpression or constitutive activation induces numerous cancers^2^. All proteins in the EGFR family are transmembrane proteins, containing an extracellular ligand binding domain, a transmembrane domain, an intracellular receptor tyrosine kinase domain, and a C-terminal signaling tail domain^6^. The members of the EGFR family share high structural homology in their tyrosine kinase domain but are distinct in their extracellular and C-terminal domains^7^. The binding of growth factors to the extracellular domain induces receptor homo- or heterodimerization and activates the kinase domain, which would lead to the autophosphorylation of the intracellular tyrosine residues at the C-terminal and the subsequent induction of downstream signals^8^,^9^. We therefore attempted to design and synthesize a series of novel EGFR tyrosine kinase inhibitors which could be beneficial to patients suffering from various cancers.

Fry *et al.*^10^ first discovered that the 4-anilinoquinazoline derivative PD153035 possesses specific inhibitory activity against EGFR tyrosine kinase. Since then, various quinazoline derivatives have been synthesized, including reversible inhibitors, such as erlotinib, gefitinib, and lapatinib, and the irreversible inhibitors BIBW2992, (*E*)-N-(4-(3-chloro-4-fluorophenylamino)-3-cyano-7-ethoxyquinolin-6-yl)-4-(dimethylamino)but-2-enamide (EKB-569)^11^. Modification of the quinazoline structure has been performed in many anticancer studies, such as against NSCLC, PC3, BTC, and MCF-7 cells^12^,^13^,^14^,^15^,^16^,^17^. Xu *et al.*^18^,^19^,^20^ envisioned that replacement of the quinazoline could lead to the identification of potent EGFR/ErbB-2 dual inhibitors.

Subsequently, in order to validate whether these designed compounds can target EGFR, the molecular docking was performed by fitting these designed compounds and reference compound (Erlotinib) into the ATP binding site of EGFR. (PDB code: 1M17). Then, the obtained results have been plotted as a line-scatter graph and presented in Fig. 1, which mainly displays the corresponding CDOCKER_INTERACTION_ENERGY of the molecular docking studies^21^. Compared with the positive drug Erlotinib, it was clearly seen that compound **3p** showed obviously lower interaction energy than Erlotinib that reached up to −48.81 kcal/mol. Besides, all the designed molecules above the cyanine dash lines possessed lower interaction energy, demonstrating that they are likely to exhibit more potent inhibitory activity against EGFR tyrosine kinase. Besides, ADMET properties are important conditions and major parts of pharmacokinetics. The ADMET predictions of the present 8 compounds showed satisfactory results (Fig. 2). Therefore, these preliminary analysis served as a stimulant to synthesize these *N-*(4,6-dimethoxypyrimidin-2-yl)-2-(piperazin-1-yl)acetamide compounds.

## Result

### MTT assay for cell viability/proliferation

To test the antiproliferative activities of the synthesized compounds (see supplementary Scheme S1 and S2), the target compounds were evaluated *in vitro* antiproliferation assays against three human cancer cell lines (MCF-7, HeLa and A549) and one human normal lung cell line. The results were summarized in Table 1. With few exception, the active analogs showed a remarkable potential antiproliferative activity, suggesting that *N-*(4,6-dimethoxypyrimidin-2-yl)-2-(piperazin-1-yl)acetamide derivatives could significantly enhance antiproliferative potency. For the given compounds, it was observed that compound **3p** showed the most potent antiproliferative activity (IC_50_ = 0.22 *μ*M for MCF-7, IC_50_ = 0.08 *μ*M for HeLa, 0.05 *μ*M for A549 and 189.2 *μ*M for Lung Cancer).

### Annexin V-FITC/PI apoptosis detection

**Figure 3:**
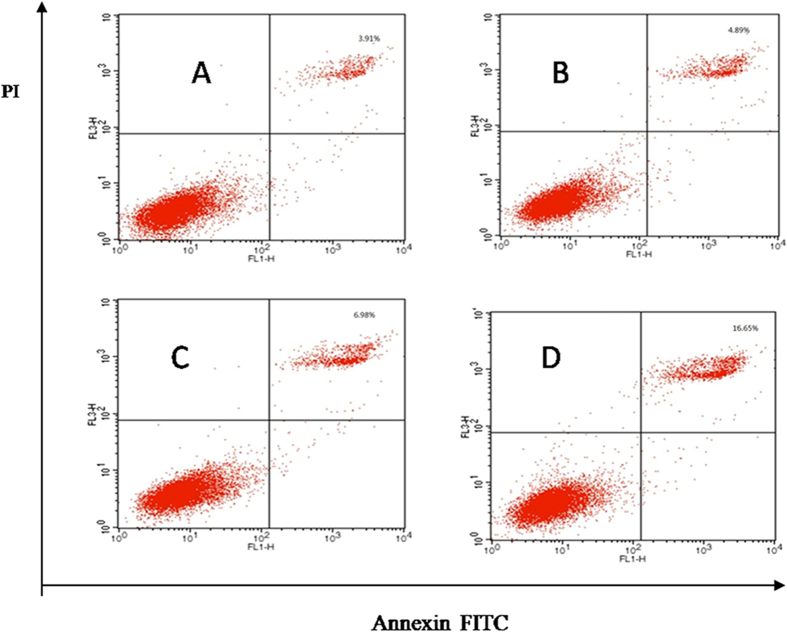
HeLa cells were cultured with anticancer and various concentrations of 3p for 24 h. Cells were stained by Annexin VeFITC/PI and apoptosis was analyzed by flow cytometry. (**A**) control. (**B**) 0.03 *μ*M. (**C**) 0.06 *μ*M. (**D**) 0.12 *μ*M.

**Figure 4:**
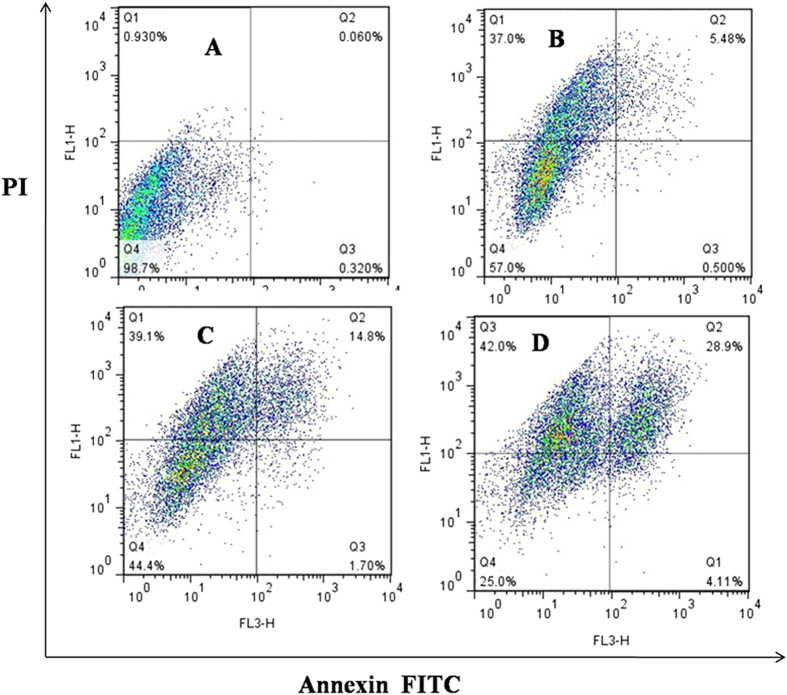
A549 cells were cultured with anticancer and various concentrations of 3p for 24 h. Cells were stained by Annexin VeFITC/PI and apoptosis was analyzed by flow cytometry. (**A**) control. (**B**) 0.03 *μ*M. (**C**) 0.06 *μ*M. (**D**) 0.12 *μ*M.

Besides, we detected the mechanism of compound **3p** inhibition activity by flow cytometry (FCM) (Figs 3 and 4) and found that the compound could induce the apoptosis of activated Hela and A549 cells in a dose-dependent manner. As shown in Figs 3 and 4, HeLa and A549 cells were treated with 0.03, 0.06 and 0.12 *μ*M of compound **3p** for 24 h. The compound increased the percentage of apoptosis by Annexin V-FITC/PI staining in a dose-dependent.

### Kinase inhibitor selectivity

**Figure 5:**
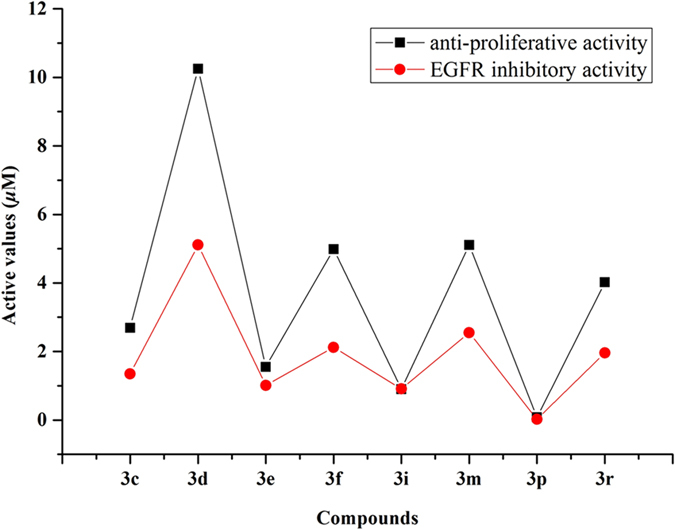
Correlation between the anti-proliferative activity against HeLa and the EGFR inhibitory activity, which indicated that there was a moderate correlation between EGFR inhibition and inhibition of cellular proliferation.

To validate whether the above anti-proliferative effect was produced by interaction of EGFR protein and the synthesized compounds, the synthesized compounds were evaluated for their abilities to inhibit the activity of four protein kinases relevant to cancer: EGFR, VEGFR2, bFGF, PDGFR. As expected, all compounds displayed the best inhibitory activity for EGFR and the results in Table 2 showed that all compounds lowered nearly one order of magnitude in the inhibition for other three protein kinases, when compared to EGFR. Additionally, the activity data inferred that the IC_50_ values of these compounds shared a similar tendency with their relevant IC_50_ values of anti-proliferative assay. Hence, a further study between the anti-proliferative activity against HeLa cell line and the EGFR inhibitory activity of these compounds was analyzed and the result indicated that there was a moderate correlation between EGFR inhibition and inhibition of cancer cellular proliferation, as evidenced in Fig. 5. The correlation coefficient *r*^*2*^ was found to be 0.979. Therefore, we could conclude that the synthesized inhibitors can inhibit the function of EGFR and the anti-proliferative effect was produced partly by interaction of EGFR protein and the compounds.

### Toxicity and evaluation of therapeutic effect *in vivo*

Acute oral toxicity was tested according to OECD guideline 423. All animals survived and appeared active and healthy throughout the study. With the exception of one male that exhibited a loss in body weight between Day 7 and 14, all animals gained bodyweight over the 14-day observation period. There were no signs of gross toxicity or abnormal behavior. Besides, we tested their cytotoxic activity on a mouse embryonic fibroblast cell line (NIH-3T3) using the MTT assay to prove the potency of the compounds^22^. The pharmacological results of these compounds were summarized in Table 3. It can be seen from Table 3 that the compounds displayed low hemolytic activities. It can be concluded that the compounds with potent inhibitory activity were low toxic, which was comparable to the positive control DDCP^23^.

**Figure 6:**
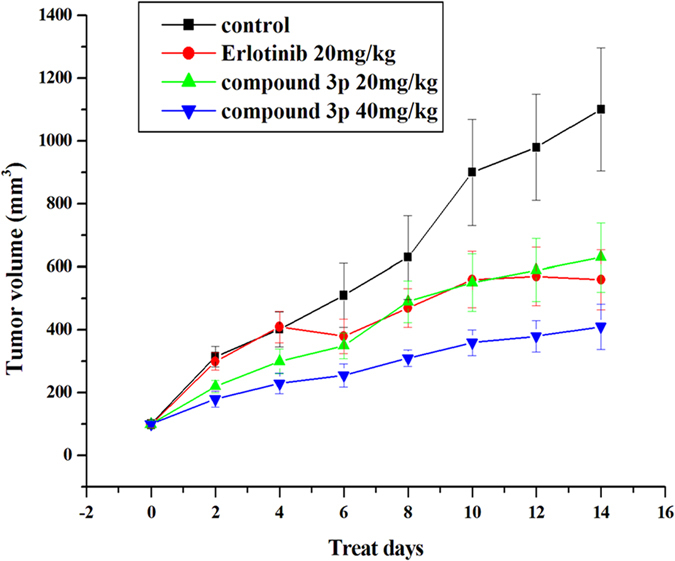
The tumor volumes in mice treated with saline, Erlotinib, compound 3p (20 mg/kg) and compound 3p (40 mg/kg).

**Figure 7:**
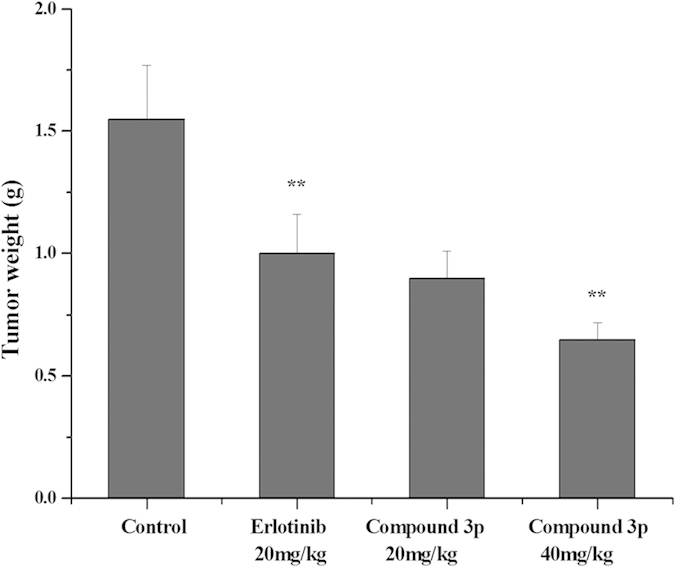
The tumor weight in mice treated with saline, Erlotinib, compound 3p (20 mg/kg) and compound 3p (40 mg/kg) at day 14.

**Figure 8:**
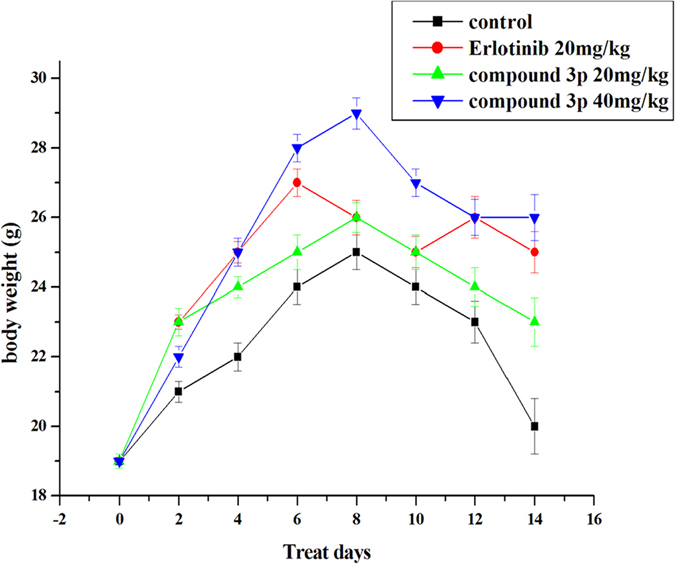
The mice weight was examined every other day.

To further evaluate the antitumor effect of compound **3p** *in vivo*, we performed an animal study. Mice were inoculated subcutaneously with A549 cells (5.0 × 10^6^) on their shoulders. The tumor volume in Erlotinib- or compound **3p**-treated mice was less than that in negative control (saline) mice at the same measurement day (Fig. 6). Values of *Test/Control* in the 40 mg/kg compound **3p** group were 42.79% (day 4), 49.61% (day 6), 50.79% (day 8), 60.00% (day 10), 61.22% (day 12) and 62.73% (day 14), indicating that compound **3p** significantly inhibited tumor growth during the 14-day treatment. The weight of tumor was also significantly reduced in mice treated with compound **3p** (40 mg/kg) (Fig. 7) and none of the mice died during the treatment. By comparison, the antitumor drug Erlotinib reduced the growth of tumors by 49.09% at day 14 in the same animal system. Meanwhile, we did not observe significant growth inhibition of mice body weight in the group of compound **3p** treatment (Fig. 8). These results indicated that compound **3p** had a significant *in vivo* antitumor activity in mice, with little effect on the normal growth of the animals.

### Docking simulations

**Figure 9:**
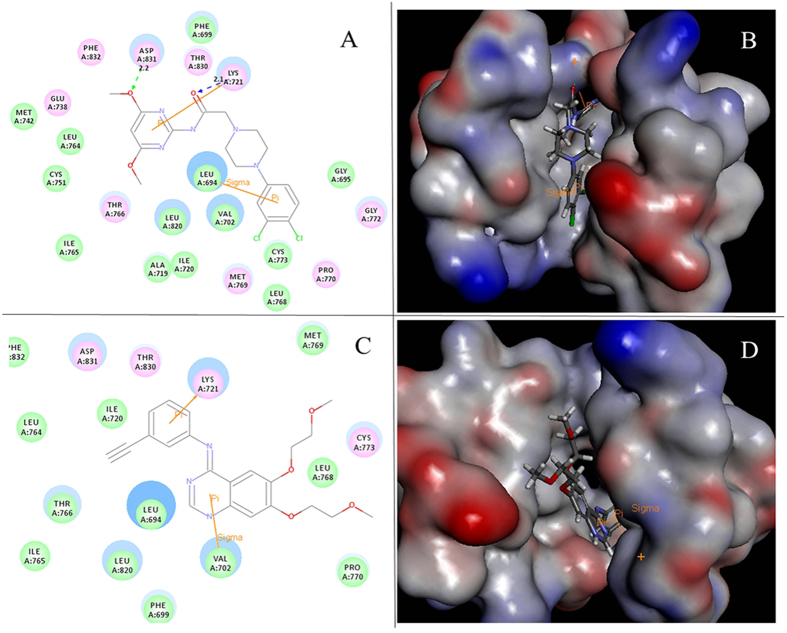
(**A**) 2D molecular docking model of compound **3p** with 1M17. (**B**) 3D interaction map between compound **3p** and 1M17 binding site. (**C**) 2D diagram of docking structure of compound erlotinib with 1M17. (**D**) 3D Model of the interaction between compound erlotinib and 1M17 binding site.

Docking study was performed to fit compound **3p** into the active center of the epidermal growth factor family (PDB code: 1M17). The obtained results were presented in Fig. 9. Figure 9A,B showed the binding mode of compound **3p** interacting with EGFR protein and the docking results revealed that three amino acids Leu694, Lys721 and Asp831 located in the binding pocket of protein played a vital roles in the conformation with compound **3p**, which were stabilized by two hydrogen bonds and *π*–sigma interaction that shown in 2D and 3D diagram. One hydrogen bond with 2.2 Å was formed between Lys721 and O of the Carbonyl group while the other hydrogen bond with 2.1 Å was involved in Asp831 and the oxygen atom on methoxy group. Figure 9C,D displayed 2D and 3D interactional maps between the original small molecule ligand Erlotinib and 1M17 protein crystal structure. Insight into those two pictures, we can see that amino acid residues Lys721, Leu694 located in the binding pocket also seemed very important for the active conformation of compound Erlotinib. These results could provide a molecular level foundation to illustrate compound **3p** can bind well at the active site of EGFR tyrosine kinase.

### 3D Quantitative Structure-Activity Relationship (QSAR) model

**Figure 10:**
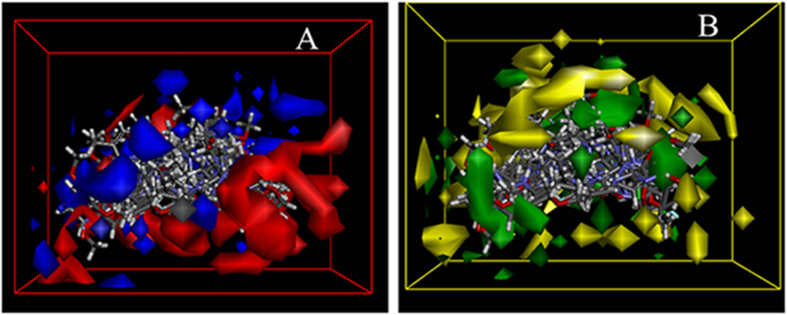
3D-QSAR of designed compounds for EGFR (PDB code: 1M17). (**A**) Isosurface of the 3D-QSAR model coefficients on electrostatic potential grids. The blue triangle mesh represents positive electrostatic potential and the red area represents negative electrostatic potential. (**B**) Isosurface of the 3D-QSAR model coefficients on Van der Waals grids. The green triangle mesh representation indicates positive coefficients; the yellow triangle mesh indicates negative coefficients.

In order to obtain a systematic SAR profile on *N*-(4,6-dimethoxypyrimidin-2-yl)-2-(piperazin-1-yl)acetamide derivatives as antitumor agents and to explore the more potent and selective EGFR inhibitors, 3D-QSAR model was built to choose activity conformation of the designed molecular and reasonably evaluated the designed molecules by using the corresponding pIC_50_ values which were converted from the obtained IC_50_ (*μ*M) values of EGFR inhibition (the way of this transformation was derived from an online calculator developed by an indian medicinal chemistry lab (http://www.sanjeevslab.org/tools-IC50.html)) and performed by built-in QSAR software of Discovery Studio 3.5 (DS 3.5, Accelrys, Co. Ltd). The training and test sets were divided by the random diverse molecules method of DS 3.5, in which the training set accounted for 83% of all the molecules while the test set was set to 17%. The graphical relationship of observed and predicted values has illustrated in Fig. 10. In which the plot of the observed IC_50_ versus the predicted values showed that this model could be used in prediction of activity for *N-*(4,6-dimethoxypyrimidin-2-yl)-2-(piperazin-1-yl)acetamide derivatives.

Also the molecules aligned with the iso-surfaces of the 3D-QSAR model coefficients on electrostatic potential grids (Fig. 10A) and Van der Waals grids (Fig. 10B) were listed. Electrostatic map indicated red contours around regions where high electron density (negative charge) was expected to increase activity, and blue contours represent areas where low electron density (partial positive charge) was expected to increase activity. Similarly, steric map indicated areas where steric bulk was predicted to increase (green) or decrease (yellow) activity. It was widely acceptable that a better inhibitor based on the 3D-QSAR model should have strong Van der Waals attraction in the green areas and a polar group in the blue electrostatic potential areas (which were dominant close to the skeleton). As expected, those potent compounds (**3p, 3i, 3e** and so on) not only could circumvent the red subregion or the unfavorable yellow steric subregion but also can get more close to the favorable blue and green spaces. Thus, this promising model would provide a guideline to design and optimize more effective EGFR inhibitors and pave the way for us in the further study.

### Single crystal *X*-ray diffraction

**Figure 11:**
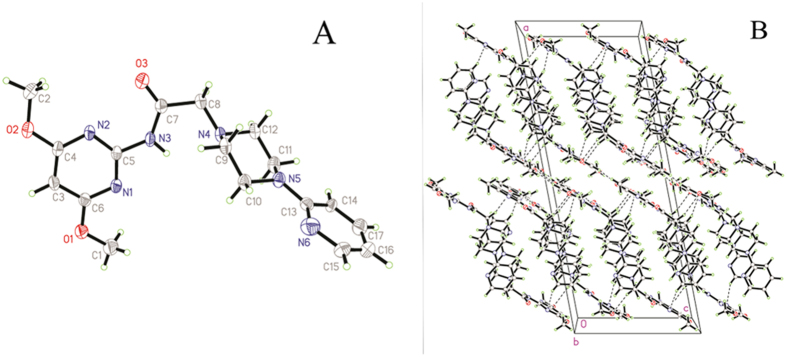
Crystal structure of compound 3m. (**A**) Molecular structure of compound **3m** with atomic numbering scheme. (**B**) Crystal packing of the compound **3m.**

Crystals of compound **3m** were obtained from methanol solution. Figure 11A shows a perspective view of the monomeric unit with the atomic numbering scheme, and Fig. 11B depicts the intramolecular and intermolecular hydrogen bonds. Crystallographic data, details of data collection and structure refinement parameters are listed in Table 4. Single crystal of **3m** (0.32 mm × 0.27 mm × 0.25 mm) was mounted on a *D*-8 venture diffractometer equipped with graphite-monochromated MoKa (*λ* = 0.71073 Å) radiation. For **3m**, a total of 8021 reflections were collected, of which 3148 were unique with R_int_ = 0.073 and 1686 observed reflections with I > 2*σ* (I) were used in the succeeding structure calculations. The final cycle of refinement of full matrix least-squares was converged to R = 0.0625 and *w*R = 0.1947. The highest and lowest residual peaks in the final difference Fourier map are 0.50 and −0.50 e/Å^3^, respectively.

## Discussion

In conclusion, a series of *N-*(4,6-dimethoxypyrimidin-2-yl)-2-(piperazin-1-yl)acetamide derivatives have been synthesized and evaluated for their antitumor activities. According to the data presented in Table 1, it could be concluded that the activity of the tested compounds may be correlated to the variation and modifications of structure. Compounds having benzhydryl substituent (**3g**, **3h** and **3o**) exhibited potent inhibitory activity, with IC_50_ ranging from 0.11 to 2.15 *μ*M. Meanwhile, a comparison of the substitution on the phenylpiperazine ring was demonstrated as follows: methoxy-substituted derivatives (**3b**, **3e** and **3i**) had better anticancer activities compared to the positive control, while the antitumor activities of halogen-substituent derivatives were decreased. Interestingly, compounds **3p** containing two substituents displayed the most potent anticancer activities among the synthesized compounds, however, compounds **3j** and **3l** containing two substituents as well, displayed poor antitumor activities. Moreover, compound **3d** had the worst anticancer activity.

Moreover, compound **3p** demonstrated the most potent inhibitory activity against EGFR with IC_50_ of 0.08 *μ*M. Docking simulation was performed to position compound **3p** into the EGFR active site to determine the probable binding conformation and the result indicated that compound **3p** was a potent inhibitor of EGFR. Besides, all of the compounds showed druglike 3D QSAR and ADMET properties. Given the unforeseen structural differences within the active site of some pathogenic enzymes, the key to discover inhibitors with antitumor activity lies in a detailed understanding of the EGFR active sites. Further studies on the EGFR inhibition ability of this compound, new structural data were guiding further modifications of the current series with the aim to improve both enzymatic inhibition and physical properties.

## Materials and methods

### Chemistry section

(The detailed information is in Supplementary Information)

### Biological section

#### Cancer cell antiproliferative assay

The *in vitro* anticancer activities of the prepared compounds against MCF-7, HeLa and A549 cell lines were evaluated as described in the literature^23^ with some modifications. Target tumor cells were grown to log phase in DMEM medium supplemented with 10% fetal bovine serum. After reaching a dilution of 1 × 10^5^ cells mL^−1^ with the medium, 100 *μ*L of the obtained cell suspension was added to each well of 96-well culture plates. Subsequently, incubation was performed at 37 °C in 5% CO_2_ atmosphere for 48 h before the cytotoxicity assessment. Tested samples at preset concentrations were added to 6 wells with Erlotinib being employed as a positive reference. After 72 h exposure period, 25 *μ*L of PBS containing 2.5 mg mL^−1^ of MTT was added to each well. After 4 h, the medium was replaced by 150 *μ*L DMSO to dissolve the purple formazan crystals produced. The absorbance at 570 nm of each well was measured with an ELISA plate reader. The data represented the mean of three independent experiments in triplicate and were expressed as means ± SD. The IC_50_ value was defined as the concentration at which 50% of the cells could survive.

#### Kinase selectivity assay

The EGFR, VEGFR2, bFGF and PDGFR Kinase Assay Kit were purchased from Bio-Swamp. The experiments were performed according to the manufacturer’s instructions.

#### Apoptosis assay

To detect the apoptosis induced by compound **3p**, HeLa and A549 cells were seeded per well in 24-well plates and were incubated overnight. Then cells were treated with compound **3p** at the three different concentrations (0.03 *μ*M, 0.06 *μ*M and 0.12 *μ*M, separately). DMSO was chosen as the negative control. After 24 h, cells were harvested for the apoptosis detection. In brief, collected cells were washed once with PBS and subsequently washed once with binding buffer, and then stained with Annexin V-FITC and propidium iodide (PI) in the binding buffer for 20 min at room temperature in the dark. Apoptotic cells were quantified using a FACScan cytofluorometer (PT. Madagasi Brosa Inc. JI. Batang Hari NO. 73, Propinsi Sumatera Utara, Indonesia) plotting at least 10,000 events per sample. To quantify the data, the frequencies in all quadrants were analyzed using flowjo software. We regarded cells in the lower right quadrant (Annexin V positive/PI negative) as early apoptotic cells, and cells in upper right quadrant (Annexin V positive/PI positive) as late apoptotic cells and necrotic cells.

### Measurement of tumor volume in nude mice

This experiment was conducted in accordance with the guideline issued by the State Food and Drug Administration (SFDA of China). The animals were housed and cared for in accordance with the guidelines established by the National Science Council of Republic China. All experimental protocols were approved by Animal Care and Use Committee of Nanjing University.

Male BALB/c nude mice, 35–40 days old and weighing 18–22 g, were supplied by Shanghai Laboratory Animal Limited Company. The mice were raised in air-conditioned rooms under controlled lighting (12 h lighting/day) and were fed with standard laboratory food and water ad libitum. Before injection into the mice, the lung cancer cells (A549) were harvested by trypsinization and washed three times with cold serum-free medium and then injected in a total volume of 0.1 mL using a 1-mL latex-free syringe (BD) within 30 min of harvest. Mice were inoculated subcutaneously with A549 cells (5.0 × 10^6^) on their shoulders. When the tumor had increased to 100 mm^3^ , the mice were equally randomized into 4 groups (with 6 mice/group): saline tumor control group; compound **3p** 20 mg/kg/2 days group; compound **3p** 40 mg/kg/2 days group; and Erlotinib 20 mg/kg/2 days positive control group. The control group received 0.9% normal saline. Tumor size was measured once every 2 days in two per-pendicular dimensions with Vernier calipers and converted to tumor volume (TV) using the formula: (ab^2^)/2, where *a* and *b* refer to the longer and shorter dimensions, respectively. The body weight of the animals was measured twice a week at the same time as the tumor dimension measurement and the mortality was monitored daily. After the treatments, all mice were killed and weighed simultaneously, and then tumor was segregated and weighed.

### Safety test section

#### Acute toxicity

Before the acute toxicity experiment, mice were stopped being served food but water was kept for 3 h. The test substances were dissolved in maize germ oil^24^. The preliminary experiment was performed as follows: Four dose levels, 100, 500, 2000, and 5000 mg/kg body weight and three mice for each level were used. We observed the death and evident toxicity in 7 days to determine the general concentration range. Then the administration doses were graded with geometric progression for ease of the calculation of LD_50_. Mice were randomly divided into groups (10 mice/group). Then the mice were weighed and the test substances of different concentrations were administered to mice in varied doses by gavage (0.1 to 0.2 mL/100 g). The control groups received maize germ oil only. After the administration, food was withheld for 2 h.

#### Cytotoxicity test

The cytotoxic activity *in vitro* was measured against mouse fibroblast NIH-3T3 cells using the MTT assay. Cells were cultured in a 96-well plate at a density of 5 × 10^5^ cells and different concentrations of compounds were respectively added to each well. The incubation was permitted at 37 °C, 5% CO_2_ atmosphere for 24 h before the cytotoxicity assessments. 20 *μ*L MTT reagent (4 mg/mL) was added per well 4 h before the end of the incubation. Four hours later, the plate was centrifuged at 1200 rcf for 5 min and the supernatants were removed, each well was added with 200 *μ*L DMSO. The absorbance was measured at a wavelength of 490 nm (OD 490 nm) on an ELISA microplate reader. Three replicate wells were used for each concentration and each assay was measured three times, after which the average of IC_50_ was calculated. The cytotoxicity of each compound was expressed as the concentration of compound that reduced cell viability to 50% (IC_50_). The results were summarized in Table 3.

#### Hemolysis test

Hemolytic activity was assayed using fresh capillary human blood. Erythrocytes were collected by centrifuging the blood three times in chilled phosphate buffered saline (PBS at 4 °C) at 1000 × g for 10 min. The final pellet was resuspended in PBS to give a 2% w/v solution. Using a microtitre plate, 100 *μ*L of the erythrocyte solution was added to dextran, PLL, stearyl-PLL or stearyl-PLL+LDL (1–1000/*μ*g/mL) in a volume of 100 mL. Samples were then incubated for 3 h and the microtitre plate was centrifuged then at 1000 × g for 10 min and the supernatants (100 *μ*L) transferred into a new microtitre plate. Hemoglobin release was determined spectrophotometrically using a microtitre plate reader (absorbance at 550 nm). Results were expressed as the amount of released hemoglobin induced by the compounds as a percentage of the total. Hemolysis test was tested according to the guide of biological evaluation of medical device (SFDA, China).

### Molecular Modeling

#### Molecular Docking Study

Molecular docking of compounds into the three dimensional X-ray structure of EGFR (PDB code: 1M17) was carried out using the Discovery Studio (version 3.5) as implemented through the graphical user interface DS-CDOCKER protocol^21^. The 3D structure of EGFR (1M17) in docking study was downloaded from Protein Data Bank. The three-dimensional structures of the aforementioned compounds were constructed using Chem. 3D ultra 12.0 software [Chemical Structure Drawing Standard; Cambridge Soft corporation, USA (2010)], then they were energetically minimized by using MMFF94 with 5000 iterations and minimum RMS gradient of 0.10. All bound waters and ligands were eliminated from the protein and the polar hydrogen was added to the proteins. Each compounds would retain 10 poses, and were ranked by CDOCKER_INTERACTION_ENERGY.

#### ADMET Prediction

Absorption, distribution, metabolism, excretion, and toxicity properties (ADMET) of the 18 novel compounds were calculated using the DS software. The aqueous solubility, blood brain barrier penetration, cytochrome P450 2D6 inhibition, hepatotoxicity, human intestinal absorption and plasma protein binding were predicted using this software.

#### 3D QSAR Study

The training sets were composed of inhibitors with the corresponding pIC_50_ values which were converted from the obtained IC_50_ (*μ*M), and test sets comprised compounds of data sets. All the definition of the descriptors can be seen in the “Help” of DS 3.5 software and they were calculated by QSAR protocol of DS 3.5. The alignment conformation of each molecule was the one with lowest interaction energy in the docked results of CDOCKER. The predictive ability of 3D-QSAR modeling can be evaluated based on 18 the cross-validated correlation coefficient, which qualifies the predictive ability of the models. Scrambled test (Y scrambling) was performed to investigate the risk of chance correlations. The inhibitory potencies of compounds were randomly reordered for 30 times and subject to leave-one-out validation test respectively. The models were also validated by test sets, in which the compounds are not included in the training sets. Usually, one can believe that the modeling is reliable, when the R^2^ for test sets is 0.767.

#### Statistical analysis

Statistical analysis was performed with SPSS Version 11.0 statistic software package. Data were expressed as means ± standard deviation (SD). Comparisons between groups were performed with analysis of non-parametric test. A value of P < 0.05 was considered statistically significant.

## Additional Information

**How to cite this article**: Sun, J. *et al.* Discovery of a series of novel phenylpiperazine derivatives as EGFR TK inhibitors. *Sci. Rep.* **5**, 13934; doi: 10.1038/srep13934 (2015).

## Supplementary Material

Supplementary Information

## Figures and Tables

**Figure 1:**
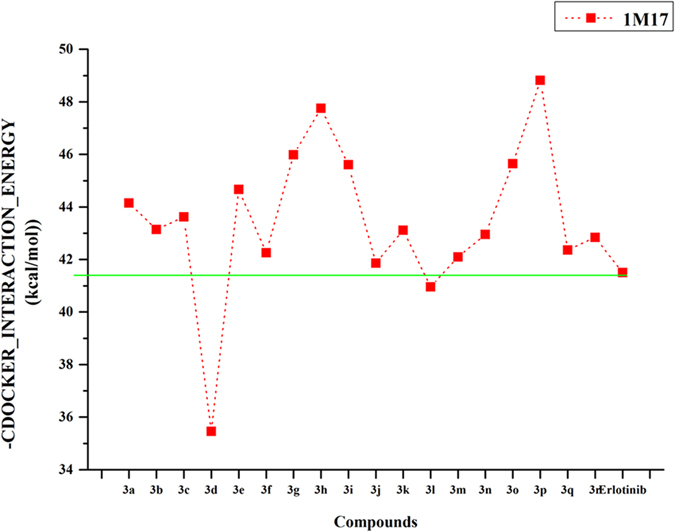
The CDOCKER_INTERACTION_ENERGY (kcal/mol) obtained from the docking study of all synthesized compounds by the CDOCKER protocol (Discovery Studio 3.1, Accelrys, Co. Ltd).

**Figure 2:**
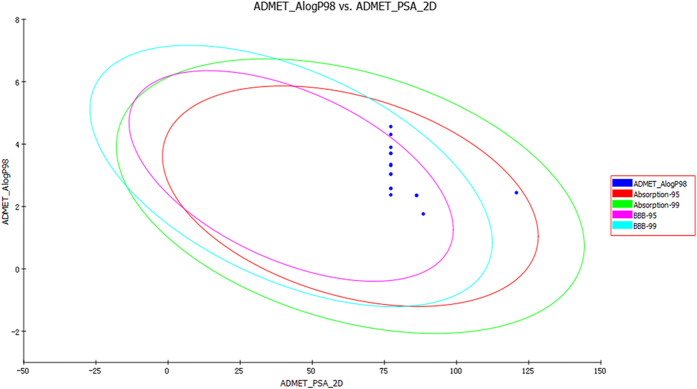
ADMET properties predicted for eighteen novel compounds. Compounds located inside the innermost oval have the best results. The eight compounds were as follows: **3m, 3r, 3f, 3p, 3c, 3d, 3e, 3i** (from top to bottom, from left to right).

**Table 1:** *In vitro* anticancer activities (IC_50_, *μ*M) against three human tumor cell lines and one human normal cell line.

compounds	R	IC_50_ ± SD (*μ*M)			
		MCF-7	HeLa	A549	Lung cells
**3c**	2-FPh	4.13 ± 0.01	2.69 ± 0.10	0.55 ± 0.02	178.2 ± 2.02
**3d**	Ph	9.87 ± 1.00	10.25 ± 0.28	2.17 ± 0.02	136.1 ± 3.15
**3e**	2-OmePh	2.57 ± 0.02	1.55 ± 0.006	1.97 ± 0.001	189.3 ± 1.78
**3f**	4-NO_2_Ph	2.09 ± 0.01	4.99 ± 0.07	0.08 ± 0.003	201.6 ± 4.05
**3i**	4-OmePh	2.98 ± 0.09	0.90 ± 0.07	2.22 ± 0.05	197.5 ± 1.74
**3m**	Py	2.87 ± 0.004	5.11 ± 0.006	1.55 ± 0.004	165.8 ± 0.59
**3p**	3,4-ClPh	0.22 ± 0.001	0.08 ± 0.0002	0.05 ± 0.0003	189.2 ± 3.82
**3r**	3-triFPh	4.97 ± 0.01	4.02 ± 0.01	0.33 ± 0.07	149.5 ± 3.03
Erlotinib	—	0.15 ± 0.03	0.11 ± 0.01	0.10 ± 0.0004	195.2 ± 1.27

**Table 2:** Inhibition of selected kinases IC_50_ (*μ*M).

compounds	EGFR	VEGFR2	bFGF	PDGFR
**3c**	1.35 ± 0.006	8.94 ± 0.73	25.37 ± 2.34	33.77 ± 1.94
**3d**	5.11 ± 0.03	10.10 ± 1.08	18.46 ± 2.11	8.50 ± 0.09
**3e**	1.01 ± 0.004	5.33 ± 0.05	11.45 ± 0.89	10.02 ± 1.17
**3f**	2.12 ± 0.08	7.67 ± 0.07	21.50 ± 0.69	24.46 ± 2.56
**3i**	0.91 ± 0.006	3.99 ± 0.08	22.35 ± 0.10	31.77 ± 3.12.
**3m**	2.55 ± 0.67	7.23 ± 0.78	3.80 ± 0.036	50.00 ± 4.76
**3p**	0.02 ± 0.003	2.15 ± 0.06	27.09 ± 3.17	17.31 ± 0.95
**3r**	1.96 ± 0.006	6.95 ± 0.68	33.94 ± 3.59	3.87 ± 0.92
Erlotinib	0.02 ± 0.003	—	—	—

**Table 3:** Hemolytic activities and cytotoxicity of these compounds.

Compounds	Hemolysis	Cytotoxicity	Bodyweights	
	LC30^a^ (mg/mL)	IC_50_ (*μ*M)	0 day (g) ± SD	7 days (g) ± SD
**3c**	>10	230.4 ± 3.15	21.09 ± 1.16	26.09 ± 0.93
**3d**	>10	150.5 ± 2.56	21.18 ± 2.15	25.57 ± 1.56
**3e**	>10	172.6 ± 5.31	21.11 ± 1.34	25.34 ± 1.15
**3f**	>10	155.3 ± 3.05	20.57 ± 1.43	27.25 ± 1.50
**3i**	>10	198.5 ± 1.13	21.33 ± 0.67	25.83 ± 1.30
**3m**	>10	182.3 ± 3.22	22.43 ± 1.02	22.09 ± 1.02
**3p**	>10	239.2 ± 4.03	22.12 ± 1.13	27.33 ± 1.08
**3r**	>10	185.3 ± 2.16	21.00 ± 1.01	25.07 ± 1.21
**DDCP**	>10	125.6 ± 3.52	21.82 ± 0.73	26.31 ± 1.22

^a^Lytic concentration 30%.

**Table 4:** Crystallographic data, details of data collection and structure refinement parameters.

compound	3m
Empirical formula	C_17_H_22_N_6_O_3_
Formula weight	358.41
Crystal system	Monoclinic
Space group	*C*_*2*_*/c*
*a* (Å)	30.984(3)
*b* (Å)	9.5308(10)
*c* (Å)	12.3680(12)
*α* (^o^)	90
*β* (^o^)	100.793(3)
*γ* (^o^)	90
*V* (Å)	3587.7(6)
*Z*	8
D calc/g cm^−3^	1.327
θ range (o)	2.2–25.1
*F*(000)	1520
Reflections collected/unique	15858/3182
Data/restraints/parameters	2151/0/237
Absorption coefficient (mm^−1^)	0.095
*R*_1_/_*W*_*R*_2_ [*I* > 2σ (*I*)]	0.0625/0.1672
*R*_1_/_*W*_*R*_2_ (all date)	0.0992/0.1947
GOOF	1.031
