# Supplementary material for: Discovery of a series of novel phenylpiperazine derivatives as EGFR TK inhibitors
Source: Sci Rep. 2015 Sep 9;5:13934. doi: 10.1038/srep13934 (PMC4563558; doi:10.1038/srep13934)
Supplement: Supplementary Information [file srep13934-s1.doc]

**Discovery of a series of novel phenylpiperazine derivatives as EGFR TK inhibitors**

Juan Sun^1,2^, Xin-Yi Wang^1^, Peng-Cheng Lv^1,2^*, Hai-Liang Zhu^1,2^*

*^1^School of Life Sciences, Shandong University of Technology, Zibo, China*

*^2^State Key Laboratory of Pharmaceutical Biotechnology, Nanjing University, Nanjing 210093, P. R. China*

** Corresponding authors.* (E-mail address: lpengc8283@163.com; zhuhl@nju.edu.cn)

**Chemistry section**

- 1. **Materials and Methods**

All of the synthesized compounds were chemically characterized by thin layer chromatography (TLC), proton nuclear magnetic resonance (^1^H NMR) and elemental microanalyses (CHN). ^1^H NMR spectra were measured on a Bruker AV-400 spectrometer at 25°C and referenced to Me_4_Si. Chemical shifts were reported in ppm (*δ*) using the residual solvent line as internal standard. Splitting patterns are designed as s, singlet; d, doublet; t, triplet; m, multiplet. ESI-MS spectra were recorded on a Mariner System 5304 Mass spectrometer. Elemental analyses were performed on a CHN-O-Rapid instrument and were within ± 0.4 % of the theoretical values. Melting points were determined on a WRS-1B apparatus (Jingke Corp., Shanghai, China) and are as read. Analytic thin-layer chromatography (TLC) was performed on the glass-backed silica gel sheets (silica gel 60 Å GF254). All compounds were detected using UV light (254 nm or 365 nm).

- 1. **General method for the preparation of target compounds**
     1. **Synthesis of 2-amin-4,6-dimethoxypyrimidine (1)**

The intermediate 2-amino-4,6-dihydroxypymidine was first synthesized with diethyl malonate and guanidine nitrate as raw materials by reaction of nucleophilic addition-elimination reaction (cyclization) in sodium ethylate. Then, 2-amin-4,6-dimethoxypyrimidine **1** was synthesized by chlorination reaction and alkoxylation reaction.

**2.2.2. Synthesis of target compounds**

Bromoacetyl bromide (1.5 mmol) was added to a stirred solution of 2-amin-4,6-dimethoxypyrimidine **1** (1 mmol) in anhydrous dichloromethane 20 (mL). The reaction mixture was left overnight at room temperature. The resulting solid 2-bromo-N-(4,5-dimethoxypyrimidin-2-yl)acetamide **2** was collected by rotary evaporation. Compound **2** (1 mmol) was dissolved in acetonitrile (20 mL) with potassium carbonate (1.5 mmol) and tetrabutylammonium bromide (0.05 mmol). The reaction mixture was refluxed for 3-5 hours and the resulting solid was collected and washed with cold ethanol, dried and crystallized from anhydrous ethanol to get the desired compounds.

**2.2.2.1**

**N-(4,6-Dimethoxypyrimidin-2-yl)-2-(4-(2-fluorophenyl)piperazin-1-yl)acetamide (3c)**

Mp: 138.5-139.7°C. ^1^H NMR (400 MHz, DMSO-*d*6): 2.860 -2.881 (m, 4H), 3.231-3.253 (m, 4H), 3.351 (s, 2H), 3.971 (s, 6H), 5.836 (s, 1H), 6.961-7.031 (m, 2H), 7.068-7.133 (m, 2H), 9.473 (s, 1H).

**2.2.2.2**

**N-(4,6-Dimethoxypyrimidin-2-yl)-2-(4-phenylpiperazin-1-yl)acetamide (3d)**

Mp: 135.2-137.4°C. ^1^H NMR (400 MHz, DMSO): 2.774 (s, 4H), 3.228-3.251 (m, 6H), 3.874 (s, 6H); 5.727 (s, 1H), 6.807-6.843 (m, 1H); 6.875-6.894 (m, 2H), 7.199-7.237 (m, 2H); 9.358 (s, 1H).

**2.2.2.3**

**N-(4,6-Dimethoxypyrimidin-2-yl)-2-(4-(2-methoxyphenyl)piperazin-1-yl)acetamide (3e)**

Mp: 139.5-140.2°C.  ^1^H NMR (400 MHz, DMSO): 2.927 (s, 4H), 3.241 (s, 4H), 3.380 (s, 2H), 3.915 (s, 3H); 3.994 (s, 6H), 5.837 (s, 1H), 6.914-6.934 (d, *J* = 8.0 Hz, 1H), 6.978-7.023 (m, 2H), 7.051-7.071(m, 1H), 9.517 (s, 1H).

**2.2.2.4**

**N-(4,6-Dimethoxypyrimidin-2-yl)-2-(4-(4-nitrophenyl)piperazin-1-yl)acetamide (3f)**

Mp: 184.8-186.7°C. ^1^H NMR (400 MHz, DMSO): 2.819-2.844 (m, 4H), 3.378 (s, 2H), 3.542-3.567 (m, 4H), 3.975 (s, 6H), 5.837 (s, 1H), 6.869-6.909 (m, 2H), 8.149-8.189 (m, 2H), 9.292 (s, 1H).

**2.2.2.5**

**N-(4,6-Dimethoxypyrimidin-2-yl)-2-(4-(4-methoxyphenyl)piperazin-1-yl)acetamide (3i)**

Mp: 120.4-124.7°C. ^1^H NMR (400 MHz, DMSO): 2.948 (s, 4H), 3.274 (s, 4H); 3.820 (s, 3H), 3.886 (m, 2H), 3.987 (s, 6H), 5.837 (s, 1H), 6.890-6.912 (m, 2H), 6.990-7.011 (m, 2H), 9.404 (s, 1H).

**2.2.2.6**

**N-(4,6-Dimethoxypyrimidin-2-yl)-2-(4-(pyridin-2-yl)piperazin-1-yl)acetamide(3m)**

Mp: 131.1-131.3°C. ^1^H NMR (400 MHz, DMSO): 2.773-2.798 (m, 3H); 3.316 (s, 2H), 3.667-3.691 (m, 4H), 3.974-3.983 (m, 6H), 5.836 (s, 1H), 6.681-6.724 (m, 1H), 7.308 (s, 1H), 7.518-7.562 (m, 1H), 8.231-8.246 (m, 1H), 9.501 (s, 1H).

**2.2.2.7**

**2-(4-(3,4-dichlorophenyl)piperazin-1-yl)-N-(4,6-dimethoxypyrimidin-2-yl)acetamide(3p)**

Mp: 153.4-154.0°C. ^1^H NMR (400 MHz, DMSO): 2.808-2.833 (m, 4H), 3.290-3.314 (m, 4H), 3.338 (s, 2H), 3.984 (s, 6H), 5.839 (s, 1H); 6.785-6.814 (m, 1H), 7.012-7.019 (m, 1H), 7.308-7.340 (m, 1H), 9.386 (s, 1H).

**2.2.2.8**

**N-(4,6-Dimethoxypyrimidin-2-yl)-2-(4-(3-(trifluoromethyl)phenyl)piperazin-1-yl)acetamide(3r)**

Mp: 136.0-136.2°C. ^1^H NMR (400 MHz, DMSO): 2.871 (s, 4H), 2.992 (s, 1H); 2.996 (s, 1H); 2.922 (s, 2H), 3.373-3.340 (m, 4H), 3.985 (s, 6H), 5.839 (s, 1H), 7.118-7.164 (m, 3H), 7.308 (s, 1H), 7.388-7.428 (m, 1H), 9.413 (s, 1H).

**Table S1. Hydrogen Bond Lengths (Å) and Bond Angles (°) of compound 3m.**

| D–H...A | d(D–H) | d(H…A) | d(D…A) | ∠DHA |
| --- | --- | --- | --- | --- |
| N(3)…H(3)…N(4) | 0.86 | 2.26 | 2.702(3) | 112 |
| C(1)…H(1)…O(3) | 0.96 | 2.46 | 3.064(5) | 120 |
| C(3)…H(3)…O(2) | 0.93 | 2.50 | 3.417(3) | 171 |
| C(8)…H(8)...O(1) | 0.97 | 2.57 | 3.468(4) | 154 |
| C(10)…H(10)…N(6) | 0.97 | 2.52 | 2.895(5) | 103 |
| C(15)…H(15)...N(2) | 0.93 | 2.61 | 3.452(4) | 151 |

**Scheme S1. Synthesis of compound 4,6-dimethoxypyrimidin-2-amine (1)**

Reagents and conditions: (i) Ethanol, sodium ethoxide, reflux, 1h. (ii) Acetonitrile, POCl_3_, reflux, 3h. (iii) H_2_O, _P_H 7, 3 h. (iv) Methanol, sodium methylate, reflux, 3 h.

**Scheme S2. Synthesis of compounds.**

Reagents and conditions: (v) Bromoacetyl bromide, dichloromethane, rt, 8 h. (vi) substituted phenylpiperazine, acetone, reflux, 5 h.
